# Biology of Lymphedema

**DOI:** 10.3390/biology10040261

**Published:** 2021-03-25

**Authors:** Bianca Brix, Omar Sery, Alberto Onorato, Christian Ure, Andreas Roessler, Nandu Goswami

**Affiliations:** 1 Gravitational Physiology and Medicine Research Unit, Division of Physiology, Otto Loewi Research Center, Medical University of Graz, 3810 Graz, Austria; bianca.brix@medunigraz.at (B.B.); andreas.roessler@medunigraz.at (A.R.); 2 Faculty of Science, Masaryk University, Kotlářská 2, 61137 Brno, Czech Republic; omarsery@sci.muni.cz; 3 Linfamed, 33100 Udine, Italy; a.onorato@linfamed.it; 4 Wolfsberg Clinical Center for Lymphatic Disorders, Wolfsberg State Hospital, KABEG, 9400 Wolfsberg, Austria; christian.ure@kabeg.at

**Keywords:** lymphatic vasculature, lymphedema, complete decongestive therapy, manual lymphatic drainage, cardiovascular system, hemodynamics, fluid shifts, perometry, plasma volume

## Abstract

**Simple Summary:**

Lymphedema is a chronic, debilitating disease of the lymphatic vasculature. Although several reviews focus on the anatomy and physiology of the lymphatic system, this review provides an overview of the lymphatic vasculature and, moreover, of lymphatic system dysfunction and lymphedema. Further, we aim at advancing the knowledge in the area of lymphatic system function and how dysfunction of the lymphatic system—as seen in lymphedema—affects physiological systems, such as the cardiovascular system, and how those might be modulated by lymphedema therapy.

**Abstract:**

This narrative review portrays the lymphatic system, a poorly understood but important physiological system. While several reviews have been published that are related to the biology of the lymphatic system and lymphedema, the physiological alternations, which arise due to disturbances of this system, and during lymphedema therapy, are poorly understood and, consequently, not widely reported. We present an inclusive collection of evidence from the scientific literature reflecting important developments in lymphedema research over the last few decades. This review aims at advancing the knowledge on the area of lymphatic system function as well as how system dysfunction, as seen in lymphedema, affects physiological systems and how lymphedema therapy modulates these mechanisms. We propose that future studies should aim at investigating, in-detail, aspects that are related to fluid regulation, hemodynamic responses, and endothelial and/or vascular changes due to lymphedema and lymphedema therapy.

## 1. Introduction

The lymphatic system is a vascular network that is more frequently researched in the past years. However, it is still far from being fully understood. This review examines the lymphatic system and lymphedema from a variety of perspectives. Limited knowledge is available regarding how lymphedema as well as lymphedema therapy affect other physiological systems, such as fluid shifts or the cardiovascular system, especially hemodynamic responses and endothelial/vascular (dys-)function. Therefore, this review systematically assesses the possible effects of lymphedema and its therapy on fluid mobilization, hemodynamic parameters at rest, and in response to orthostatic loading, as well as vascular function.

## 2. Overview of the Lymphatic System

### 2.1. Anatomy

The lymphatic system includes a variety of structures and so-called lymphoid organs, including the spleen, thymus, and tonsils, all having their specialized role in, e.g., immune function. However, this review focuses on the lymphatic vasculature, which is—compared to the blood vasculature—a unidirectional transport system. It starts at the peripheral capillary beds of the blood vessels and it runs throughout the whole body [1], organized as vascular tree, and it can be anatomically separated into different sections dependent on the location (distal to central) [1]: (i) initial lymphatic vessels; (ii) collector lymphatic vessels (pre- and post-nodal); (iii) lymph nodes; and, (iv) lymphatic trunks. For detailed anatomy of the lymphatic system, please see [1,2,3,4,5,6,7].

### 2.2. Physiology

In order to fulfil its role in providing fluid and nutrients to the different tissues, blood vessels continuously leak plasma and proteins at the capillary bed / microvasculature into the interstitial space. This mechanism is driven by an imbalance in hydrostatic and osmotic pressure, which is widely known as “Starling equation” [8]. Approximately eight liters of plasma are filtered each day [8]. The accumulation of excessive fluid in the interstitial space generates pressure. This is the driving force for fluid to enter into the initial lymphatics via primary valves [9,10]. As the initial lymphatics lie very close to the microvasculature, they serve as entry point for lymphatic fluid [1].

Two primary forces are responsible for “pushing” lymphatic fluid trough the lymphatic vessels: extrinsic forces, e.g., muscle movement, heart contraction or respiration, as well as intrinsic forces. The anatomical structures of the vasculature, specifically the lymphangions, are of importance in this process. Along with muscle contraction, this enables the vasculature to work as a pump. Lymphatic fluid is pumped from each lymphangion to the next. Structural segments further support this process in the form of unidirectional valves that are made of endothelial cells and connective tissue at each end of the lymphangions. Their main role is to prevent backflow of lymphatic fluid. This is particularly important while standing in an upright position, as the lymph flux must be driven against gravity [1]. Sequential peristaltic as well as segmented contractions of lymphangions, together with the lymphatic valves, prevent backflow [11,12]. This active, intrinsic pump mechanism plays a crucial role in the regular flow within the lymphatic system [13]. Neuronal, humoral, and mechanical stimuli (e.g., pressure or shear stress) can enhance fluid flow and optimize lymph function [14]. Similar to blood vessels, lymphatic vessels also express flow-mediated nitric oxide (NO) production [15]. Scallan et al. (2013) suggested a potential exchange mechanism between lymphatics and tissues, as atrial and brain natriuretic peptides have shown to modify the permeability of collecting lymphatics [16]. Lymph fluid consists of immune cells, proteins, lipids, lipoproteins, electrolytes, and bacteria (including potential harmful compounds). Passing through the lymphatic vasculature, lymph fluid is directed through at least one lymph node. There, an adequate immune answer eliminates bacteria and any potentially harmful particles. Thus, blood vessels that are located within the nodes transport different compounds, such as fluid, proteins, and cells [1]. Additionally, lymphatic fluid is mechanically filtered within lymph nodes. This allows protein-free fluid to pass through the blood–lymph barrier. Therefore, afferent lymph can be concentrated by the reabsorption of water [17], which leads to a higher protein concentration in post-nodal lymphatic fluid [18]. From the eight liters of lymphatic fluid that is produced every day, reabsorption processes in the lymph nodes reduce the efferent outcome to about four liters [19]. Finally, Gannon and Carati (2003) reported the expression of Aquaporin-1 water channels in lymph nodes in an animal model and, thus, suggest that a transcellular transport of water might contribute to protein concentrations [4,20].

### 2.3. Methods for Assessing Lymphatic Flow

Lymphoscintigraphy is used as a diagnostic tool to image lymphatic vessels. It includes the injection of radioactive colloid particles. The accumulation of those within the lymphatic vasculature and lymph nodes can then be determined. It is used as the gold standard to determine whether tissue swelling occurs due to lymphatic dysfunction [21]. However, more suitable to show lymphatic flow is the use of near-infrared fluorescence dyes, in particular indocyanine green (ICG). This allows a quantitative analysis of lymph flow [22]. Another option is magnetic resonance lymphangiography, which is highly suitable for visualizing lymphatic vessels, but scintigraphy is superior in detecting lymph nodes [23,24]. Near infrared fluorescence imaging uses fluorescent dyes to visualize lymphatic vessels. The fluorescent dye indocyanine green is primarily bound to albumin and, due to its large molecular size, it is restricted to the lymphatic vessels. This technique is routinely used in lymphatic-venous anastomosis surgery to find suitable vessels [21]. Lipidiol has been used as contrast medium in transpedal lymphangiography, as it is retained within the lymphatic vessels when compared to other agents, which tend to diffuse out of the vessels more rapidly [25]. Further, dynamic contrast-enhanced magnetic resonance lymphangiography (MLR) is a technique that has been used for imaging central conducting lymphatic vessels, using T1-weighted MR images. This method has been applied in imaging as well as treatment planning of lymphatic leaks, thoracic duct, and other lymphatic abnormalities, as well as chyloperitoneum and chylothorax [25,26]. Finally, a novel, innovative tool for the visualization of the lymphatic vasculature is a 3D imaging technique known as a multispectral optoacoustic tomography device (MSOT). This method allows for the precise detection of lymphatic vessels in real-time, utilizing a hand-held device. It has shown to be especially useful in the detection of deep lymphatic vessels that could not be detected via ICG lymphography [27]. The readers are referred to Polomska et al. (2020) for further details on lymphatic imaging [28].

Hyaluronic acid (HA), a biogenic macromolecule, is transported into the blood stream solely via the lymphatic vessels [29]. The level of plasma HA reflects a dynamic balance between delivery to and the bloodstream removal. Therefore, HA has recently been proposed as a novel biomarker in the assessment of lymphatic flow [29,30,31]. Previously, increased plasma hyaluronic acid levels have been reported, e.g., during exercise [32] or post-prandial in healthy subjects [33]. Roh et al. (2017) showed that the hyaluronic acid levels are increased within the lymphedema tissue in a mouse model [30]. This was also observed in human lymphedema tissue [34]. In a pilot study, where we assessed HA levels before and after three weeks of CDT, we could not see changes in plasma HA (as an indicator of lymphatic outflow). These findings raise the question of whether hyaluronan in plasma can serve as a practical lymphatic outflow measure in patients with lymphedema [35]. Because of its large molecular size, HA molecules may be preserved within the lymphedematous tissue of such patients, possibly leading to the recurrent accumulation of fluid [36]. It is possible that physical therapy mobilizes fluid from the interstitial space, but without accompanying HA molecules in lymphedema patients. Hyaluronic acid could be resistant to degradation and wash out by application of local physical pressure, as, e.g., during manual lymphatic drainage. Indeed, it has been suggested that recombinant hyaluronidase [30] or local heat therapy [37] in mouse models could be effective in breaking down high molecular weight hyaluronic acid.

## 3. Overview of Lymphedema

Any imbalance in lymphatic fluid generation, transport, outflow, or dysfunctional vessels can lead to lymphedema. Lymphedema is a progressive disease, leading to a massive accumulation of fluid. It usually occurs in the extremities and it is a disabling disease that can arise due lymphatic dysfunction and can display a huge psychological burden [38]. Independent of the precise etiology, lymphedema is characterized by severe swelling, atrophic skin changes, secondary infections, and localized pain [39]. 

### 3.1. Epidemiology and Etiology

Lymphedema has been reported to often be underestimated or misdiagnosed [40]. A number of differential diagnosis, such as lipedema or venous diseases, need to be taken into account [41]. Further, only a limited number of medical doctors specialize in this field. When considering these aspects, patients often find themselves as medical nomads [40], passing several doctors until a diagnosis is made. Thus, the exact number of patients suffering from lymphedema can only be estimated. These estimates range between 140 Million and up to 300 Million people worldwide [38,42,43,44,45]. Maclellan et al. (2015) found that 25% of 255 patients included were diagnosed with “lymphedema”, although they suffered from different diseases, as e.g., venous stasis (7%), lipedema (6%), or vascular malformations (1%). On average, 7.7 years passed between disease onset and the allocation to a treatment program. About 70% of the patients in this study population were female [40]. Neuhüttler and Brenner (2006) also showed that females are more likely to develop lymphedemas when compared to males (females:males; 4.6:1) [46]. The results from a bigger, multi-center investigation that was performed in several countries (France, Turkey, United Kingdom, Canada, and more) are similar: 79% of the patients included were women [47].

While not yet completely elucidated (precise pathophysiological processes underlying this disorder, its heterogeneity, root cause), it can be differentiated between primary and secondary forms. Primary lymphedema is associated with a strong genetic background (inherited or caused by spontaneous mutation), whereas secondary lymphedema is typically acquired and it evolves e.g. when lymphatic impairment occurs.

#### 3.1.1. Primary Lymphedema

Primary lymphedema is strongly associated with a genetic background in 25–30% of all primary lymphedema patients [5]. The prevalence is relatively rare, the disease is seen in one from 100.000 individuals [42]. Typically, this form of lymphedema occurs due to function impairment, which leads to a structural and/or functional dysfunction that negatively influences the drainage abilities [48]. More than 20 different genes (i.e., VEGFR-3, CCBE1, FOXC2, GATA2, GJC2, PTPN14, SOX18, CCBE1, FAT4, ADAMTS3, FBXL7, GJC2, KIF11, ITGA9, REEKIN, PIEZO1, EPHB4, CALCRL, and CELSR1) have been associated with anomalies in the lymphatic system, leading either to underdeveloped lymphatic structures or poor lymphatic outflow abilities [6,49]. Aspelund et al. (2016) reported that vascular endothelial growth factor-C (VEGF-C) and the endothelial growth factor receptor-3 (VEGFR-3) signaling axis have been found to be involved in around 50% of all primary lymphedema cases [50]. Another example is lymphedema distichiasis occurring due to mutations in the FOXC2 gene, leading to malformed lymphatic valves [51,52].

Although a genetic background in primary lymphedema has been researched in recent investigations, the disease was historically classified into three groups depending on the age of the patients, when first symptoms occurred [43]. Different sub-groups were characterized as follows [53]:

(a) congenital lymphedema: <1 year;
(b) lymphedema praecox: 1–35 years (most common sub-group); and,
(c) lymphedema tarda: <35 years.

The exact numbers of patients suffering from primary lymphedema is not known; however, it is estimated to be up to 10% higher in females when compared with males

#### 3.1.2. Secondary Lymphedema

Secondary lymphedema is the far more common form of lymphedema. The prevalence of the secondary form is estimated at one in 1000 individuals. The average age of patients at diagnosis in secondary lymphedema is between 50–58 years [42]. When compared to primary lymphedema, secondary lymphedema develops due to tissue damage, vessel obstruction or deranged lymph nodes, and/or lymphatic vessels that were intact before. Therefore, it is acknowledged as an acquired disease. Surgery, trauma, obesity, or infections (*wuchereria bancrofti*) can be the reason for this damage leading to the development of the disease [42,54]. Furthermore, it often occurs following cancer treatment, e.g., radiotherapy or the dissection of lymph nodes [42,53]. Breast cancer related lymphedema (BCRL) is the most common form of secondary lymphedema. The risk of developing lymphedema after breast cancer treatment was reported to be about 15–20% [48]. A systematic review and meta-analysis of BCRL cases shows an overall incidence of 15.5% after cancer treatment [55]. Bar Ad et al. (2010) reported that 16% of all patients undergoing treatment with a combination of lymph node resection and radiation therapy developed clinical signs of lymphedema [56], whereas radiation therapy led to the highest incidences at 31% [55]. Radiation therapy can lead to the reduction of proliferative potential of the lymphatic tissue, fibrosis of the lymph nodes or to a mechanical insufficiency of the lymphatic vasculature [57]. Additional co-factors in the development of secondary lymphedema can be congestive heart failure or renal diseases [58]. It has further been suggested that several genes (e.g. *VEGFR2*, *VEGFR3*, *RORC*, *GJC2*, and *FOXC2*) are possibly involved in secondary lymphedema developing following breast cancer therapy [59,60,61].

#### 3.1.3. Lymphatic Malformations and Chylous Effusions

Lymphatic malformations (LMs) are defined as congenital slow-flow vascular malformations and they are characterized by lesions in the lymphatic vessels, consisting of dilated lymphatic vessels and cystic-like areas that are filled with lymphatic fluid [62,63]. They are typically found in the head and neck area as well as pelvis and axilla. Depending on the appearance, they are categorized as macro-, micro-, and mixed-cystic. LMs can be diagnosed by medical history, physical examination, as well as ultrasound and MRI. Additionally, the lymphatic endothelium marker Poloplanin D2-40 may facilitate diagnosis [62,63]. Masthoff et al. (2018) reported that MSOT may be a promising tool in diagnosing lymphatic malformations, since it has been shown to distinguish other types of vascular malformations [64].

During surgery, trauma of lymphatic vessels may result in a postoperative leakage of lymphatic fluid. Different kinds of these postoperative lymphatic leakages have been described, e.g., lymphatic ascites, lymphorrhea, chylothorax, chylous ascites, or chylorrhea [65]. Chylous thoracic or abdominal effusions can, for example, be treated via transinguinal lymphangiography using lipoidol [66]. Further, thoracic duct embolization (TDE) has shown to be an alternative treatment option for chylous leaks. A high rate of success, together with minimal occurrence of complications, have been reported [67].

### 3.2. Clinical Assessment of Lymphedema

Diagnosis of lymphedema can be challenging due to the high variabilities in etiology and underlying mechanism in the development of lymphedema and due to the fact that it is not as easy to be differentiated from other forms of edema. This often leads to lymphedema being underdiagnosed and undertreated. The variable way by which lymphedema is clinically diagnosed and defined is a confounding attribute in the diagnosis process [38,68,69]. However, it is important to diagnose and treat lymphedema as soon as possible, as the early detection of the disease is crucial for therapy approaches and outcome, as well as for limiting disease progression. A recent study including 149 patients showed that early detection, along with a simple intervention (which was up to six weeks of self-massage by the patient and usage of a compression garment), can be highly effective in patients at risk of BCRL [70]. Unfortunately, early stages of lymphedema cannot always be easily differentiated from other causes, leading to edema, as e.g., obesity or venous diseases [39]. Schook et al. (2011) reported that, in 27% of pediatric patients, other anomalies, such as post-traumatic swellings or lipedema, are mistaken for lymphedema [71]. Standard diagnostic tools do not usually enable the diagnosis of lymphedema at a subclinical stage.

Although several genes and biomarkers that are associated with lymphedema have been investigated [72,73], up to now most of the diagnoses are performed via clinical history and examination, including aspects that are related to family history and carrying out detailed physical examination involving palpation and skin inspection [39,74]. In the patients’ history, crucial aspects include, e.g., cancer treatment or trauma as well as signs of typical symptoms (swelling, skin changes, and recurrent infections). The Stemmer sign is one of the most reported clinical signs in diagnosing the presence of lymphedema. If it is not possible to pinch a skinfold on the second toe, it is considered positive [75]. In a study, lymphatic function was tested using lymphoscintigraphy in patients showing a positive Stemmer sign. The results from this study indicated that a positive Stemmer sign is indeed a reliable predictor for lymphedema [75]. Approximately 15% of the patients show skin changes, such as hyperkeratosis or lymphorrhea [71]. Other typical symptoms, which are often described by patients is the feeling of heaviness in the affected limb. Together with acute swelling, this is associated with a positive predictive value for the presence of lymphedema [73].

Especially in early stages, diagnosis of lymphedema can be challenging as changes in limb volume or circumference are not detectable. Tape measurement, water displacement, perometry, and bioelectrical impedance spectroscopy (BIS) are diagnostic tools with good reliability and validity. BIS is the only method that is currently available to detect stage I lymphedema. BIS enables the assessment of whole body composition as well as segmental composition (e.g., of the limbs). This method uses an electrical current to distinguish between total body water, extracellular, and intracellular fluid volumes based on the tissue resistances [76]. Tape measurement can be highly observer dependent. Therefore, perometry represents a more reliable tool with increased interobserver-reliability [77]. Imaging techniques (lymphoscintigraphy, lymphangiography, or computer tomography) are used to detect lymphatic flow impairment as an underlying mechanism [39].

### 3.3. Therapeutic Options

There is currently no definite cure available for lymphedema. However, various approaches have been investigated and they are currently researched. Ciudad et al. (2019) suggest that the treatment of lymphedema should start as early as possible. The reason for this is that progression of lymphedema into higher stages leads to fibrotic tissue changes and this destruction of possible remaining functional lymphatic vessels [78]. All of the following therapeutic approaches have a common goal: the reduction of accumulated fluid in the respective body part, by promoting and/or developing alternative pathways from deranged lymphatic structures to tissues where intact lymphatic flow properties can be found [79]. This serves the overall aim of improving functional status [78] and preventing disease progression [80].

#### 3.3.1. Physical Therapy

Physical treatments include massages, lymphatic drainages, the application of different kind of compression garments, and intermittent pneumatic compression [81]. Natural compression can be simulated by intermitted pneumatic compression devices, which use a sequential airflow to inflate special hoses and, therefore, applying positive pressure on the tissue. A meta-analysis study showed its efficacy in patients that were diagnosed with secondary lymphedema [82]. It can be applied either alone or in combination with other physical therapies [82].

Gott et al. (2018) have described a novel therapeutic approach that uses the application of negative pressure (representing a pulling/opening force). While this approach has already been used in the field of wound healing, its use in lymphedema has not yet been fully investigated [83]. Another form of negative pressure uses kinesiology tapes. These tapes are used to decongest lymphatic fluid that accumulates under the skin. It is additionally recommended to compression bandages, for body parts where compression garments cannot be easily fitted and in cases where bandages could be uncomfortable [84]. A meta-analysis carried out showed that the quality of life (QoL) is higher in patients under treatment with kinesiological methods. However, the efficacy of this method was not proven in this study [84]. Davies et al. (2020) reported that the use of kinesiology tapes leads to the reduction of volume. However, they also state that this form of treatment is not superior when compared to other interventions [85].

Fascia manipulation [86,87], weight loss [43,88,89,90], heat treatment [91], or extracorporeal shock wave therapy [92] and far infrared radiation treatment [93] have also been used as therapeutical approaches. Currently, these approaches are mainly used in research, and are, therefore, not routinely applied. Further investigations in bigger clinical studies are needed. Please see Tzani et al. (2018) for further details on different physiotherapeutic approaches in lymphedema patients [81].

#### 3.3.2. Innovative Drugs / Compounds for the Treatment of Lymphedema

Diuretics were believed to have a beneficial effect in some lymphedema patients. However, imbalance of electrolytes and fluid can occur with its usage and, therefore, administration is generally not recommended [79]. Benzopyrones may positively influence lymphatic fluid absorption by assisting in hydrolyzing proteins and activating the route of lymphatic transport [79]. However, this has not yet been confirmed in clinical studies. It did not improve limb volume or quality in life in lymphedema patients and, additionally, long-term administration can lead to adverse effects on the liver [94]. Another compound, Ketoprofen, reduced lymphedema in a mouse model. Ketoprofen is an anti-inflammatory agent [95,96]. Rockson et al. (2018) investigated the effect of anti-inflammatory therapy in an open-labeled and placebo-controlled study. They reported decreases in the thickness of the skin, decreases in the expression of granulocyte-colon-stimulating-factor-1 (GCSF), and changes in the histopathology of lymphedema tissue [95]. Decreased levels of GCSF, as seen following ketoprofen administration, might have a beneficial effect [95]. Further investigations are required to confirm these beneficial effects because this was only shown in a pilot study.

T-cells are believed to play a role in the development of lymphedema as they inhibit lymphatic angiogenesis and aid in tissue fibrosis. Therefore, Tacrolimus has been tested as immunosuppressive drug sensitive to CD4^+^ T-cells in animals [97]. This compound is also widely used in transplantation medicine in humans [98]. In terms of lymphedema, applied locally, it was able to prevent the formation of lymphedema, but also showed to be effective once lymphedema was already established. Again, this has only been investigated in animal models and clinical studies are required to confirm these results and provide additional information on the safety of this compound in humans [97]. 

Already in the early 1960s, a number of researchers reported the idea of using hyaluronidase treatment in lymphedema and deforming lymphedema stage III [99,100,101]. In an animal model of induced lymphedema, Roh et al. (2017) used hyaluronidase, which was injected into the lymphedema tissue. This led to the reduction in the limb with acquired lymphedema. They suggest that lymphangiogenesis is promoted by hyaluronidase and further, HA fragment size is modified by the enzyme. As low molecular weight hyaluronic acid has shown to promote LYVE-1 expression (a player in lymphangiogenesis), it was postulated that regulating HA fragment size might also modulate mechanism of lymphedema [30]. Therefore, this could potentially be used as a novel therapy in lymphedema patient. This, however, still needs to be confirmed and tested in clinical studies [30].

Another approach that has been investigated is VEGF-C delivery through gene therapy or injection. Animal studies suggest that VEGF-C delivery enhances lymphangiogenesis as well as the lymphatic pump function via a VEGF receptor 3 dependent mechanism [102], and it was able to reduce lymphedema [103,104]. A recent report has shown that Lymfactin^®^, a combined adenoviral VEGF-C, in combination with lymph node transfer, is well tolerated in a phase I clinical trial including upper limb lymphedema patients [105].

Further, mesenchymal stem cells showed beneficial effects by differentiating into lymphatic endothelial like cells in several clinical studies [44,106,107,108]. However, no changes in arm volume were found in a follow up study (up to one year) as well as no significant improvement, being assessed via lymphoscintigraphy post-treatment with adipose-derived stem cells [109]. Hu et al. (2020) report a new promising approach in secondary lymphedema treatment by using adipose-derived stem cells; however, this still needs to be evaluated in larger clinical studies [110].

Overall, it appears that drug treatments / medications are not yet able to provide a safe and effective therapy for lymphedema. However, several approaches and compounds being actively used in animal and clinical studies could potentially provide innovative therapy options in the future [111]. Finally, surgical options are utilized if conservative therapy approaches do not show the expected successful outcomes (see [44,78,112]).

#### 3.3.3. Surgical Approaches

More recently, innovative surgical procedures have been investigated in animal models and clinical studies. These are based on different factors, for example, advances in the field of microsurgery as well as novel insights into the lymphatic vasculature itself, and a better understanding of the underlying pathophysiology of lymphedema [78]. The surgical interventions can be separated into physiological and excisional procedures. Whereas, physiological procedures are aimed at promoting fluid flow properties, either by redirecting the lymphatic flow directly into the venous system or by providing new pathways, excisional procedures involve the removal of affected tissue parts. Table 1, below, outlines the different surgical approaches in lymphedema.

Lymphvenous Anastomosis (LVA) and vascularized lymph node transfer (VLNT) are the two most performed surgeries in lymphedema patients. During LVA surgery, a connection between the lymphatic structures and blood vessels is established. Although patients report an improvement in symptoms after LVA, applying compression garments is still required post-surgery. Evidence suggests that LVA is more effective in the upper limbs as compared to the lower extremities [78]. VLNT is performed in more advanced stages in lymphedema patients in which lymphatic vessels are dysfunctional and/or lymph nodes are not present. A range of donor sites have been described in the literature: Jejunal, gastroepiploic, ileocecal, appendicular, or supraclavicular lymph nodes. Different factors, e.g., prior exposition to radiation, scars, aesthetic appearance, or disease stage, are taken into account when choosing the recipient site [112]. Two theories on the underlying mechanisms have been suggested [78]: (1) accumulated fluid in the close area is absorbed by the nodes; and, (2) VEGF-C induced lymph angiogenesis by vascularized lymph nodes LVA and LVNT have both shown promising outcomes in clinical studies in terms of limb volume reduction and reduced episodes of cellulitis. However, no beneficial effect in reducing fibrosis has been observed. Additional future studies are required to evaluate the long-term outcomes of these interventional approaches [44].

In cases of fibroadipose hypertrophy, as can often be found in chronic lymphedema, suction assisted lipectomy (SAL) can be the surgery of choice. As this procedure shows minor improvement of lymphedema, wearing compression bandages lifelong is necessary in order to not risk recurrence [44]. Although the overall outcome is very much dependent on the patient and their strict following of the recommendations after surgery, its efficacy in long-term reduction of limb volume has been shown in different studies [78].

Radical reduction of lymphedema with perforator preservation is a mixture of physiological and excisional surgery, and while some studies show positive and long-term results, it needs outstanding microsurgical abilities, and it has a greater risk of scar forming, risk of contamination, necrosis, and longer operational times when compared to the above-mentioned approaches. It is typically used in advanced stages of lymphedema (stage III) [78]. The Charles procedure implies the complete removal of the skin and subcutaneous tissue from the impacted area and is performed to minimize excessive thickness and prevent inflammation, particularly in patients with advanced stage severe lymphedema. However, it shows very poor long-term results and is, therefore, obsolete [78].

## 4. Complete Decongestive Therapy

Up to date, there is no cure for lymphedema (except obesity induced lymphedema [89,113]). Although several therapeutic options, surgical and non-surgical, have been tested and are currently researched, the therapy of choice is a form of physical therapy that is known as complete/complex decongestive (physio-)therapy (CDT). This form of treatment is not curative for lymphedema, but mainly aims at reducing fluid volume as well as preventing the disease from progression [68].

### 4.1. Underlying Principles

CDT is an empirically driven and multicomponent therapy program [68]. It can generally be separated into two phases. Phase one involves manual lymphatic drainage (MLD), usage of multilayered compression bandages, carrying out physical exercises, and meticulous skin care. The second phase mainly focuses on self-care via elastic sleeves or compression stocks application as well as continuous exercise [114]. The main components of CDT include:

- *Manual Lymphatic Drainage (MLD)* is performed to enhance lymphatic outflow. Lymph therapists use specific hand movements (rhythmic, flowing or stirring) with a pressure of 30—40 mmHg in a frequency that mimics the intrinsic frequency of the lymphangion (10/min). MLD is started in the area of healthy tissue and then expanded into adjacent areas where the obstructed vessels are located [115,116].
- *Compression bandages* are applied, aiming at increasing interstitial pressure and therefore, to decrease capillary filtration [117] leading to a decrease in accumulated fluid/volume [115,116].
- *Physical exercise* such as ergometry [118], aerobic exercise [119] and/or resistance exercise [120] as well as associated respiratory movements are believed to assist in increasing lymphatic flow, in reducing swelling and in improving muscle strength as well as quality of life in lymphedema patients [82].
- *Skin care and skin restauration* [121].
- *Psychological support* [38] as the disabling and debilitating characterizations from lymphedema are a huge psychological burden to the patient.
- *Educational seminars* on skin care or nutrition [122].

### 4.2. Research into the Effects of Physical Therapy

As described above, the main aim and, therefore, outcome parameter of CDT is the reduction of overall volume and circumference of the affected part of the body, accompanied by improvements of functionality and quality of life. Several studies compare volume and circumference changes before and after different forms of therapy [114,123], with different durations reaching from six days intensive therapy [124] up to several months of treatment [70,125,126]. Water displacement [127], perometry [128], and tape measurement of the limb circumference [129] are the standard methods for these assessments. Apart from the key aspects of CDT (MLD and compression), modified treatments, such as other compression garments or duration and/or composition of therapy, have been investigated [91,130,131,132]. Overall, there appears to be a lack of existing literature related to the amount of fluid, which is mobilized by CDT, where this fluid is shifted to, and how the body responds to the additional mobilized fluid. Table 2 provides an overview of the effects of lymphedema per se as well as lymphedema therapy, specifically physical therapy, as in complete decongestive treatment: what is known, the knowledge gaps and how they can potentially be addressed.

#### 4.2.1. Studying Fluid Shifts Caused by Treatment

As described above, the reduction of overall volume and circumference of the affected part of the body, accompanied by improvements of functionality and quality of life, is the main aim and, therefore, outcome parameter of CDT. Questionnaires are mostly used to assess changes in quality of life of the patients [114,123]. Water displacement [127], perometry [128] and tape measurement of the limb circumference [129] are the standard methods for these assessments. Overall, there appears to be a lack of existing literature related to the amount of fluid, which is mobilized by the CDT, where this fluid is shifted to, and how the body responds to, the additional mobilized fluid. Bioelectrical impedance analysis (BIA) or Bioelectrical impedance spectroscopy (BIS) are methods that enable not only the diagnosis of lymphedema, by calculating the ratio between a healthy and affected leg, but distinguishing between extracellular and intracellular fluids [133]. Using BIS, Pereira de Godoy et al. (2013) determined how much fluid is mobilized following seven days of intensive physical therapy in patients with lower limb lymphedema [134]. They observed decreases in total water, but increases in intracellular water in the affected limb. They interprete their findings as indicative of fluid mobilization from the affected limb to healthy parts of the body, as they observed increases the total water in the trunk and upper extremities [134]. A more recent study from the same research group was only partially able to confirm these results, as they found a reduction of intracellular and extracellular water following therapy in the legs and increased levels of intra- and extracellular water, in the trunk and upper limbs [124]. However, these findings were only assessed prior- and post- physical therapy of seven days. Recently, we tested the hypothesis that fluid mobilization or fluid shifts to different parts of the body induced by CDT will be reflected in plasma volume changes (PVC) and plasma composition. As lymphedema therapy (CDT) mobilizes lymphatic fluid outflow—which means a greater return of lymphatic fluid into the blood circulation—an increase in plasma volume at the level of amount of fluid mobilized from the lymphatic system should be expected. As different equations have been previously used to calculate relative changes in plasma volume, we utilized them in our study. To calculate the relative change in plasma volume, each of these equations uses different blood sample parameters. For example, some equations are based on changes in plasma density [135], hematocrit [136], hematocrit and hemoglobin [137], and both anthropometric data and hematocrit [138]. While most of these equations calculate relative changes in plasma volume [135,136,137], the equation according to Nadler [138] estimates absolute changes in plasma volume. Changes in plasma volume, especially central hypervolemia, are associated with changes in plasma protein (total protein, albumin, and albumin/globulin ratio), oncotic pressure, electrolyte concentrations (sodium, chloride, and potassium), osmolality, volume regulating hormones, and blood pressure. We observed that fluid shifts occur due to physical therapy and manual lymphatic drainage [139]. Manual lymphatic drainage affects the limbs and whole-body fluid composition. Fluid shifts due to manual lymphatic drainage are also reflected in plasma volume increases as well as plasma protein increases. All four formulae for plasma volume changes calculations led to similar results. This could be an indirect indicator of the concentration and composition of lymphatic fluid entering into the blood stream through the thoracic duct. Because plasma volume increases due to physical therapy could be associated with compensatory hemodynamic—and volume regulatory hormonal—responses, these aspects should be examined in future studies.

#### 4.2.2. Studying Lymphedema Treatment Effects on Orthostatic Intolerance

Sodium homeostasis has been shown to play an important role in blood pressure regulation [5]. Animal studies have shown that lymphatic vessels as well as macrophages contribute to both blood pressure control and balance of interstitial fluid [140]. Sodium is stored in the skin, where it is found bound to proteoglycans without water retention [141]. Further investigations have shown that VEGF-C and VEGFR-2 also have some role in blood pressure regulation [142], as the accumulation of sodium together with a higher blood pressure was seen upon inhibition of those [5]. When patients displaying refractory hypertension symptoms were compared to controls with normal blood pressure levels, higher concentrations of VEGF-C were found in the plasma [143]. This could be based on the following hypotheses [5]: the lymphatic system facilitates enhanced interstitial sodium clearance, which is controlled by kidney excretion, and/or that VEGFR-2 mediates the production of endothelial nitric oxide (NO) production and, therefore, vasodilation.

Orthostasis describes an upright posture, as during standing. During an upright posture, the cardiovascular system is affected by gravitational forces, leading to a reduction in blood pressure, as a certain amount of blood is pooled in the lower body. If the cardiovascular system is not able to counteract this blood pressure drop, this can lead to symptoms of fainting, a loss of consciousness, known as syncope, as the mean arterial pressure cannot be stabilized [144,145]. This can be a major problem in people having a history of feeling dizzy when standing up or also in older people. Syncope occurs due to the inability of the cardiovascular system to maintain a certain level of the mean arterial pressure (MAP) during an orthostatic challenge (standing up), which further leads to a critically reduced cerebral blood flow. Hormonal factors [146,147], issues in cerebral autoregulation [148], dysfunction within the autonomic system [149], or cardiac problems [150] are only a few examples of the factors that are involved in the etiology of syncope.

##### The mechanisms of Falls: Role of Cardio-Postural Interactions and Medications

The supposedly simple task of standing up, the transition from a supine or sitting to a standing position, contributes to approximately 40% of all falls [151]. While age associated muscle function and structural changes in the muscles (sarcopenia) contributes to falls in the elderly, increasing numbers of falls due to orthostatic hypotension are seen. Deranged motor control has been reported as the most important reason that is involved in this mechanism. Further key factors involved are cardio-postural regulation and cerebral perfusion [152]. Additionally, medications, such as diuretics or anti-hypertensive therapy, have been known to cause falls [153]. Therefore, the increased risk of collapse after medication is related to impaired cardio-postural and deranged cerebral perfusion. This also needs to be considered when medications are prescribed for lymphedema patients.

##### Alteration of Cardio-Postural Interactions during Lymphedema Treatment?

Different external and internal stimuli can affect posture in everyday life and, therefore, imposes a risk of stable standing balance. The ability to detect disturbances in posture and to react accordingly is required to maintain this balance. Age plays an important role here. It has been noted that these abilities are negatively correlated with age, as they deteriorate with higher age, and could, therefore, lead to imbalance and an increased risk of falls [154,155,156,157]. The higher occurrence of falls because of postural hypotension, together with the loss of postural stability, is of major concern not only in older people, but also in patients. Similar changes in cardio-postural control and blood pressure regulation may also occur in patients with lymphedema due to different fluid volumes in the legs, especially when comparing pre - to post - treatment in these patients.

Esmer et al. (2019) investigated the hemodynamic responses to MLD different body regions. They observed a reduction in systolic blood pressure after MLD in the neck, abdomen, and the lower limbs. Heart rate seemed to decrease after massage in the arms. Diastolic blood pressure showed decreased values after MLD in the neck and legs, but increased after abdominal MLD. Esmer et al. (2019) demonstrated different acute responses of the hemodynamic system, depending on the body part treated by MLD [158]. However, they only investigated the effects due to MLD on blood pressure, but no cardio postural interaction was assessed during MLD and throughout the several weeks of CDT.

Cardio-postural interaction is a combination of postural sway, electromyography of the legs (EMG), blood pressure (BP), and electrocardiogram (ECG) measurements. This could be investigated by a simple sit-to-stand test [159,160]. Based on this model, we have developed a system to collect, integrate, and analyze these signals to provide an integrated approach of assessing interactions between the cardiovascular and postural control [150,161] during therapy. This blood pressure regulation and cardio-postural responses to orthostatic challenge could differ in patients with lymphedema as compared to healthy controls, due to the amount of fluid, which is mobilized during therapy. Research should be carried out in the future regarding how lymphedema influences cardio-postural interactions.

#### 4.2.3. Vascular/Endothelial (dys-)function

Atherosclerosis is a chronic inflammatory disease, which involves the inner layer of the arterial wall. Plaques are built within the inner lumen of the vessels. These lead to the narrowing of the artery. Atherosclerosis is a disease that develops over several years or decades. Two essential processes are a part of disease development: the recruitment of immune cells and accumulation of cholesterol [50]. How these two aspects work together in this process is not yet fully elucidated. However, what is known is that the removal of cholesterol from the endothelium of the vessel has been directly linked to the regression of the disease [162]. A process known as reverse cholesterol transport (RCT) and, further, high-density lipoprotein (HDL) formation, is involved in this removal [163]. The lymphatic vasculature was only recently associated with the transport of HDL from the interstitial space into the blood stream [50,164,165]. In a study by Lim et al. (2013), VEGF-C administration enhanced lymphangiogenesis and further lead to reduced levels of cholesterol through an improvement of RCT. Moreover, in surgically induced lymphedema, a reduction of RCT of about 20% was observed [164]. Similar to this, a reduction of 77% in RCT was found in a model with no dermal lymphatic structures [165]. Taking this into account, the lymphatic vessels are reported to be actively involved in lipoprotein metabolism and cholesterol levels in the plasma. Moreover, the lymphatic vasculature is of importance for RCT function [5,166,167].

As part of atherosclerosis, endothelial dysfunction can occur, and it is a main predictor of cardiovascular diseases. Endothelial cells are not only the inner layer of the vascular lumen, but they also contribute to blood flow properties and, therefore, blood pressure regulation. Endothelial cells can either release vasoconstrictors, such as endothelin (ET) or platelet-activating factor (PAF), or vasodilators, such as nitric oxide (NO) or prostacyclin (PGI2). Different factors can stimulate further NO production via the endothelial nitric oxide synthase (eNOS) [168]. One of these important factors is the shear stress on the vessel wall [169]. During standing up or during exercise, shear stress is increased and it stimulates an increased NO production [170]. These increased levels of NO result in a flow-mediated dilatation (FMD) in large arteries. Endothelial dysfunction, and in specific NO reduction, is thought to be a driving factor in hypertension pathogenesis [171], atherosclerosis, and other cardiovascular disorders [172]. If endothelial dysfunction is detected at an early stage, then it can be reversed via different clinical approaches.

We have previously reported that lymphedema therapy affects endothelial function in lymphedema patients undergoing three weeks of complete decongestive therapy [173]. Methods for studying endothelial and vascular function are flow-mediated dilatation (FMD), EndoPAT2000, pulse wave velocity (PVW), and retinal imaging. All of these are non-invasive and easy to use also in patients. Flow mediated dilatation (FMD) is an ultrasound-based measurement that can be used to determine brachial artery vascular reactivity after occluding and releasing blood flow [174,175]. Endothelial vasodilator function can be assessed by measuring changes in volume using probes in the fingertips. Arterial stiffness can be investigated by measuring pulse wave velocity (PWV). Blood pressure cuffs are applied at different parts of the body to e.g., determine carotid-femoral (PWVcf) or brachial-ankle pulse wave velocity (PWVba) [176]. Another innovative and unique opportunity to assess retinal microvasculature changes via retinal fundoscopy. This method can be used to analyze retinal arterioles and venules characteristics from a single picture taken with a fundus camera. Arteriolar-to-venous ratio, vessel diameter, and vessel tortuosity index can be measured [177,178,179,180].

## 5. Conclusions and Recommendations

This review summarizes the current knowledge that is related to the lymphatic system and examined the lymphatic system and lymphedema from a variety of perspectives. While several reviews have been published related to the biology of the lymphatic system and lymphedema, the physiological alternations, which arise due to disturbances of this system (e.g., in lymphedema), and during lymphedema therapy, are poorly understood (Table 2). Therefore, there is a need for future investigations aiming at studying in-detail aspects that are related to fluid regulation/mobilization, hemodynamic responses, and endothelial and/or vascular changes and how they are altered in lymphedema as well as due to lymphedema therapy.

## Figures and Tables

**Table 1:** Surgical approaches applied in lymphedema patients.

Physiological Procedures	Excisional Procedures
Lymphvenous Anastomosis (LVA)	Suction Assisted Lipectomy (SAL)
Vascularized Lymph Node Transfer (VLNT)	The Charles procedure
Radical reduction with preservation of perforators	

**Table 2:** Overview of lymphedema and lymphedema therapy, current knowledge, knowledge gaps, and how those can potentially be addressed.

Lymphedema		Complete Decongestive Therapy (CDT)
Fluid accumulation in limbs Chronic inflammatory state Associated with risk factors leading to cardiovascular diseases	Current knowledge	CDT mobilizes fluid from the lymphedematous tissue
How the excess fluid accumulation in the lower limbs affects cardio-postural control Does lymphedema influence endothelial/vascular (dys-)function?	Knowledge gaps	Effect of CDT on mobilized fluid unclear Distribution of mobilized fluid not known (intra– and extracellular body fluid, blood circulation, lymphatic system) Effects on plasma volume changes Effects on plasma content (proteins, electrolytes) Effects on hormonal responses How do fluid shifts affect hemodynamics and blood pressure regulation? How does fluid shift affect cardio-postural control → falls? Effect of CDT on endothelial / vascular function?
Assess hemodynamic responses (blood pressure, heart rate, cerebral blood flow) to orthostatic loading (via sit-to-stand test) Vascular function assessments (Pulse wave velocity, flow mediated dilatation), including microvasculature assessment (retinal microvasculature analysis)	How to address these knowledge gaps	CDT effects on mobilized fluid can be assessed via perometry and bioelectrical impedance spectroscopy (distribution of fluid shifts), Plasma volume changes can be calculated using different formulae (based on e.g., plasma density, hematocrit, hemoglobin) Plasma protein and electrolyte concentrations, osmolality, oncotic pressure and hormonal measurements Sit-to-stand test to monitor cardio-postural control pre – and post - therapy Vascular function assessments (Pulse wave velocity, flow mediated dilatation), including microvasculature assessment (retinal microvasculature analysis) over three weeks of therapy
Cardio-postural control could be impaired in those patients → orthostatic intolerance → increased risk of falls Impaired endothelium/vascular function could increase cardiovascular risk in lymphedema patients	Why addressing the knowledge gaps is important.	CDT effects on mobilized fluid could potentially lead to postural hypotension and falls Manual lymphatic drainage could also affect cardio-postural control and blood pressure regulation leading to falls Vascular / endothelial (dys-) function need to be assessed over several weeks of therapy to assess beneficial effects of physical therapy
Falls are associated with chronic hospitalization and increased costs Signs of endothelial (dys-) function can be reversed if detected early	Long-term impacts	Minimize risk of falls during therapy, thus reducing hospitalization duration, cost of care and improving quality of life Cardiovascular risk is reduced if the treatment improves or prevents deterioration of endothelial dysfunction

## Data Availability

Not applicable.
